# Survey of *Toxoplasma gondii* and *Neospora caninum*, haemotropic mycoplasmas and other arthropod-borne pathogens in cats from Albania

**DOI:** 10.1186/1756-3305-7-62

**Published:** 2014-02-11

**Authors:** Cornelia Silaghi, Martin Knaus, Dhimiter Rapti, Ilir Kusi, Enstela Shukullari, Dietmar Hamel, Kurt Pfister, Steffen Rehbein

**Affiliations:** 1 Chair of Comparative Tropical Medicine and Parasitology, Faculty of Veterinary Medicine, Ludwig-Maximilians-Universität München, Leopoldstraße 5, 80802 Munich, Germany; 2 Merial GmbH, Kathrinenhof Research Center, Rohrdorf-Lauterbach, Germany; 3 Universiteti Bujqësor, Fakulteti i Mjeksise Veterinare, Kodër Kamëz, Tirana, Albania

**Keywords:** Cat, Albania, *Toxoplasma gondii*, *Neospora caninum*, *Leishmania infantum*, *Anaplasma* spp., *Bartonella henselae*, haemotropic mycoplasmas

## Abstract

**Background:**

Albania is a country on the western part of the Balkan Peninsula. The Mediterranean climate is favourable for the stable development of many arthropod species, which are incriminated as vectors for various agents. Recently, several papers have reported on epidemiological aspects of parasitic diseases including vector-borne disease agents of dogs with zoonotic characteristics in Albania. However, data on the epidemiology of feline parasitic and bacterial agents in Albania is scarce.

**Methods:**

Serum and EDTA-blood samples collected from 146 domestic cats from Tirana during 2008 through 2010 were examined for exposure to *Toxoplasma gondii*, *Neospora caninum*, *Leishmania infantum*, and *Anaplasma* spp. with IFAT, for infection with *L. infantum*, *A. phagocytophilum, Bartonella* spp. and haemotropic mycoplasmas with conventional PCR and real-time PCR and for *Dirofilaria immitis* with antigen ELISA. Additionally blood smear microscopy was carried out for detection of blood-borne pathogens.

**Results:**

Antibodies to *T. gondii* (titre ≥1:100) were demonstrated in 91 cats (62.3%). Antibodies to *N. caninum* (titre ≥1:100), *L. infantum* (titre ≥1:64) and *Anaplasma* spp. (titre ≥1:100) were found in the serum of 15 (10.3%), 1 (0.7%) or 3 (2.1%) cats, respectively. DNA of haemotropic mycoplasmas was detected in the blood of 45 cats (30.8%), namely *Candidatus* Mycoplasma haemominutum (21.9%), *Mycoplasma haemofelis* (10.3%), and *Candidatus* Mycoplasma turicensis (5.5%), with ten cats harbouring co-infections of two mycoplasmas each; blood from one cat was PCR positive for *Bartonella henselae*. No DNA of *Leishmania* spp. and *A. phagocytophilum* or circulating *D. immitis* antigen was detected in any cat sample. The overall prevalence of haemotropic mycoplasmas was significantly higher in male compared to female cats (40.6% vs. 24.1%, p = 0.0444); and age was associated positively with the prevalence of antibodies to *T. gondii* (p = 0.0008) and the percentage of haemotropic mycoplasma infection (p = 0.0454).

**Conclusions:**

With the broad screening panel including direct and indirect methods applied in the present study, a wide spectrum of exposure to or infection with parasitic or bacterial agents was detected.

## Background

Albania is a country on the western part of the Balkan Peninsula that extends to the south from central Europe into the Mediterranean Sea. The country is bordered by Montenegro to the northwest, Kosovo to the northeast, Former Yugoslav Republic of Macedonia to the east and Greece to the south and southeast. Albania has a coast on the Adriatic Sea to the west and on the Ionian Sea to the southwest, and is less than 75 km from southern Italy, across the Strait of Otranto. The climate is Mediterranean on the Adriatic coast, but the mountainous interior of Albania has a humid continental climate with frosty and snowy winters, especially in the north-eastern part of the country.

The Mediterranean Basin is considered as a region where zoonoses are widespread and most numerous as regards variety, but often receives insufficient attention to specifically assess its situation. Dogs especially, but cats too, may act as reservoirs of pathogens of zoonotic concern as populations of dogs and cats are numerous in both urban and rural areas. Among other factors, the mild Mediterranean climate is favourable for the stable development of many arthropod species, which are incriminated as vectors for various agents [1,2].

Due to its history, Albania can be considered as a relatively uncharted country with respect to canine and feline diseases, including infections of zoonotic importance. Recently, several papers reported on aspects of the epidemiology of parasites including vector-borne disease agents of dogs with zoonotic character in Albania [3-7]. The knowledge regarding cats, however, is limited to preliminary data on parasites of internal organs, arthropod ectoparasites and the detection of zoonotic bacterial pathogens in fleas collected from cats [8-10].

The objective of this study was to assess exposure to or infection with *Toxoplasma gondii* and *Neospora caninum*, as well as bacterial and parasitic arthropod-transmitted pathogens, in serum and blood samples of cats from the capital Tirana by serological and molecular detection methods.

## Methods

### Animals

EDTA-blood and serum samples were collected from 146 (59 male, 87 female) clinically normal, free-roaming cats from suburban areas from Tirana, Albania, on four occasions either in summer (June/July, 75 cats) or autumn (October/November, 71 cats) during 2008 to 2010. The age of the cats ranged from approximately three months up to five years. The cats were categorized into the following age groups: kittens, <6 months (n = 33); juveniles, >6 and up to 12 months (n = 39); young adults, >12 and up to 36 months (n = 43); adults, >36 months (n = 31). Blood samples were frozen after collection with the exception of a total of 82 samples. Examination of the cats for ectoparasites revealed infestation with 1 to 50 fleas on 71 cats (total 320 *Ctenocephalides felis* and 5 *Ctenocephalides Ctenocephalides canis*), and a single female *Rhipicephalus sanguineus* tick was found on one other cat. As reported previously, the fleas collected from those cats and another seven cats were analyzed for the presence of flea-borne zoonotic pathogens, i.e. *Bartonella* spp. and *Rickettsia* spp. [10].

### Serology

Sera were tested for the presence and the level of IgG antibodies against *T. gondii*, *N. caninum*, *Anaplasma phagocytophilum* and *Leishmania infantum* by indirect fluorescent antibody test (IFAT). A commercial test available for cat serum was used for *T. gondii* with controls and conjugate provided in the kit (MEGASCREEN® FLUO TOXOPLASMA *gondii* Test Kit antifeline). *N. caninum* and *A. phagocytophilum* were also tested with commercial kits, [MEGASCREEN® FLUO NEOSPORA c., MEGASCREEN® FLUO ANAPLASMA ph. (MegaCor, Hörbranz, Austria)] with FITC labeled anti-cat IgG conjugate (Sigma-Aldrich, Taufkirchen, Germany). For anti-*L. infantum* antibody detection, an in-house IFAT was used with *L. infantum* promastigote stages derived from cell culture serving as antigen [11]

In the absence of validated IFAT for felines, visible cytoplasmatic/membrane fluorescence (*N. caninum, L. infantum*) or fluorescence of cytoplasmatic morulae (*A. phagocytophilum*) was considered as seroreactive.

Two-fold serial dilutions were made starting from 1:25 (*T. gondii*, *N. caninum*, *A. phagocytophilum*) or 1:32 (*L. infantum*). Reactions at a dilution of ≥1:64 for *L. infantum* or ≥1:100 for *T. gondii*, *N. caninum*, and *A. phagocytophilum* were considered positive; positive serum was serially diluted to 1:800 (*N. caninum*, *A. phagocytophilum*), 1:1600 (*T. gondii*), or 1:512 (*L. infantum*). Positive control sera for *T. gondii* were provided by the manufacturer of the test kit and by Dr. Sándor Hornok (Institute for Parasitology and Zoology, Faculty of Veterinary Medicine, Szent Istvan University, Budapest, Hungary) who also provided a *N. caninum*-positive cat serum sample. For *Leishmania* and *Anaplasma*, feline serum samples obtained from the routine diagnostic centre of the Institute of Comparative Tropical Medicine and Parasitology in Munich, with titres of >1:256, were used as positive controls.

The DiroCHEK® Canine/Feline Antigen Test Kit (Synbiotics Corp., San Diego, USA) was employed for the detection of circulating female adult heartworm proteins (*Dirofilaria immitis*).

### Direct pathogen detection and molecular biological analysis

A Giemsa-stained blood smear from each cat was microscopically examined for intra- and extracellular pathogens, and the Knott’s test was used to detect microfilariae in a subset of 82 blood samples, which had not been frozen after sampling.

DNA was extracted from the EDTA–blood with a commercial kit according to the manufacturer’s instructions for blood (QIAamp DNA MiniKit, Qiagen, Hilden, Germany). Quality and quantity of the extracted DNA were checked with a spectrophotometer (NanoDrop®1000, Peqlab, Erlangen, Germany).

Conventional PCRs were carried out for detecting DNA of *Bartonella* spp. [12], *Mycoplasma haemofelis* and *Candidatus* Mycoplasma haemominutum [13]. Species identification can be carried out according to species-specific length of the PCR-products. Primers and PCR conditions used are listed in Table 1. The HotMaster Taq DNA Polymerase Kit (5PRIME, Darmstadt, Germany) was used. For the specific detection of DNA of *L. infantum*[14], *A. phagocytophilum*[15] and *Candidatus* Mycoplasma turicensis [16] real-time PCR were carried out in an AB7500 (Applied Biosystems, Darmstadt, Germany) using the TAQMAN®GENEEXPRESSION MasterMix (Applied Biosystems, Darmstadt, Germany), according to the instructions of the manufacturer and with primers and probes under conditions summarized in Table 1. Positive and negative controls were included in each PCR run. The products of conventional PCRs were examined under UV-light, after 2% agarose gel electrophoresis and staining with GelRed™ (Biotium, Hayward, USA).

**Table 1:** Summary of specific PCR and real-time PCR methods used in this study on cats from Albania

**Target**	**Primers 5′-3′**	**Cycle conditions**	**Reference**
Conventional PCR methods			
*Bartonella* spp. 16S-23S ITS (154–260 bp) *B. henselae:* 172 bp	Barhen1_for: YCTTCGTTTCTCTTTCTTCA	44 cycles: 30 Sec 94°C, 30 Sec 60°C, 30 Sec 72°C	[12]
	Barhen2_rev: AACCAACTGAGCTACAAGCC		
*Mycoplasma haemofelis/Candidatus* M. haemominutum *16S rRNA* gene (274 bp/202 bp respectively)	OHOK1_for: ATGCCCCTCTGTGGGGGATAGCCG	35 cycles: 45 Sec 94°C, 45 Sec 58°C, 45 Sec 72°C	[13]
	CaB2_for: CTGGGAAACTAGAGCTTCGCGAGC		
	OOCBr1_rev: ATGGTATTGCTCCATCAGACTTTCG		
Real-time PCR methods			
*Leishmania infantum Kinetoplast ~700 bp*	Lsh-kF: CTTTTCTGGTCCTCCGGGTAGG	All Real-time PCR methods: 2 min 50°C, 10 min 95°C, 40 cycles: 15 Sec 95°C, 1 min 60°C	[14]
	Lsh-kR: CCACCCGGCCCTATTTTACACCAA		
	Lsh-kp: FAM-TTTTCGCAGAACGCCCCTACCCGC-BHQ1		
*Anaplasma phagocytophilum: Msp2* gene (77 bp)	ApMSP2f: ATGGAAGGTAGTGTTGGTTATGGTATT		[15]
	ApMSP2r: TTGGTCTTGAAGCGCTCGTA		
	ApMSP2p-FAM: TGGTGCCAGGGTTGAGCTTGAGATTG		
*Candidatus* M. turicensis *16S rRNA* gene (85 bp)	HS Real2_for: GAAGGCCAGACAGGTCGTAAAG		[16]
	HS Real2_rev: CTGGCACATAGTTWGCTGTCACTTA		
	HS RealT: FAM-AAATTTGATGGTACCCTCTGA-MGB		

### Statistical analysis

Exact 95% confidence intervals (95% CI) for the percentages of seropositivity or prevalences of pathogens were calculated with the Clopper and Pearson method. Fisher’s exact test or chi-squared statistics were used to test for associations of the analytical results with gender and age of the cats, season of sample collection or between analytical results themselves. P-values <0.05 were considered as significant.

This study was approved by the ethical committee of the Universiteti Bujqësor, Fakulteti i Mjeksise Veterinare, Tirana, Albania.

## Results

A total of 63.7% of the 146 cats tested positive in the indirect detection tests, as well as 31.5% with direct test methods, resulting in 72.6% of the 146 cats with evidence for exposure to *T. gondii*, *N. caninum*, *L. infantum* and/or *Anaplasma* spp., and/or infection with *Bartonella henselae* or haemotropic mycoplasmas.

### Serological examination

Ninety-one out of 146 feline serum samples (62.3%, 95% CI 53.9 - 70.2) were found positive (IFAT titre ≥1:100) for *T. gondii* (Table 2). For 14 other cats, borderline titres of 1:50 were obtained. Further titration of the 91 IFAT positive serum samples showed that 76 of the 91 (85.7%, 95% CI 76.8 - 92.2) seropositive cats had highly reactive titers of 1:800 or higher.

**Table 2:** **Details of cats that tested seropositive (IFAT titre ≥1:100) for *Toxoplasma gondii***

	**Number of cats studied**	**Number of cats seropositive for *Toxoplasma gondii*, total (%)**					
		** Titre ≥1:100**	**Titre 1:100**	**Titre 1:200**	**Titre 1:400**	**Titre 1:800**	**Titre ≥1:1600**
Male cats	59	34 (57.6, 95% CI^1^ 44.1-70.4)	0	2 (3.4)	5 (8.5)	8 (13.6)	19 (32.2)
Female cats	87	57 (65.5, 95% CI 54.6-75.4)	1 (1.2)	1 (1.2)	6 (6.9)	11 (12.6)	38 (43.7)
Kitten, ≤6 months	33	12 (36.4, 95% CI 20.4-54.9)	0	1 (3.0)	3 (9.1)	3 (9.1)	5 (15.2)
Juvenile, >6-12 months	39	23 (59.0, 95% CI 42.1-74.4)	0	0	2 (5.1)	4 (10.3)	17 (43.6)
Young adult, >12-36 months	43	30 (69.8, 95% CI 53.9-82.8)	1 (2.3)	2 (4.7)	4 (9.3)	4 (9.3)	19 (44.2)
Adult, >36 months	31	26 (83.9, 95% CI 66.2-94.6)	0	0	2 (6.5)	8 (25.8)	16 (51.6)
Total	146	91 (62.3, 95% CI 53.9-70.2)	1 (0.7)	3 (2.1)	11 (7.5)	19 (13.0)	57 (39.0)

^1^95% CI = 95% confidence interval.

Antibodies (IFAT titre ≥1:100) to *N. caninum* were found in 15 of 146 cats (10.3%, 95% CI 5.9 - 16.4), with titres of 1:100 in nine cats (6.2%), 1:200 in five cats (3.4%), and 1:400 in one cat (0.7%) (Table 3). Borderline IFAT titres of 1:50 were obtained for a further 40 cats, with 28 and seven of them showing *T. gondii* IFAT titres of ≥1:800 or 1:50 to 1:400, respectively, while the serum of the remaining five cats did not show reactivity to *T. gondii*.

**Table 3:** **Details of cats that tested seropositive (IFAT titre ≥1:100) for *Neospora caninum***

	**Number of cats studied**	**Number of cats seropositive for *Neospora caninum*, total (%)**			
		** Titre ≥1:100**	**Titre 1:100**	**Titre 1:200**	**Titre 1:400**
Male cats	59	6 (10.2, 95% CI^1^ 3.8-20.8)	6 (10.7)	0	0
Female cats	87	9 (10.3, 95% CI 4.8-18.7)	3 (3.4)	5 (5.7)	1 (1.1)
Kitten, ≤6 months	33	2 (6.1, 95% CI 0.7-20.2)	1 (3.0)	1 (3.0)	0
Juvenile, >6-12 months	39	5 (12.8, 95% CI 4.3-27.4)	2 (5.1)	2 (5.1)	1 (2.6)
Young adult, >12-36 onths	43	5 (11.6, 95% CI 3.9-25.1)	4 (9.3)	1 (2.3)	0
Adult, >36 months	31	3 (9.7, 95% CI 2.0-25.8)	2 (6.5)	1 (3.2)	0
Total	146	15 (10.3, 95% CI 5.9-16.4)	9 (6.2)	5 (3.4)	1 (0.7)

^1^95% CI = 95% confidence interval.

In addition, one (0.7%, 95% CI, 0.02-3.76) and three (2.1%, 95% CI 0.4-5.9) serum samples tested positive for antibodies to *L. infantum* (IFAT titre 1:256) or *A. phagocytophilum* (IFAT titres 1:100, 1:200 or 1:800), respectively. Borderline 1:32 anti-*L. infantum* or 1:50 anti-*Anaplasma-*IFAT titres were observed in ten and eight cats, respectively. All ELISA tests were negative for circulating *D. immitis* antigen.

### Direct pathogen detection

The blood of 1 out of 146 cats was positive for DNA of *B. henselae* (0.7%, 95% CI, 0.02-3.76). DNA of three haemotropic mycoplasmas was detected in the blood of 45 cats (30.8%): *Candidatus* M. haemominutum in 32 cats (21.9%, 95% CI 15.5-29.5), *M. haemofelis* in 15 cats (10.3%, 95% CI 5.9-16.4), and *Candidatus* M. turicensis in eight cats (5.5%, 95% CI 2.4-10.5). Ten cats harboured co-infections of two mycoplasmas each (Table 4).

**Table 4:** Details of cats that tested positive for haemotropic mycoplasmas

	**Number of cats studied**	**Number of cats tested positive for mycoplasmas, total (%)**					
		**Any mycoplasma**	***Mh***^**1**^	**C** ***Mh***^**2**^	**C** ***Mt***^**3**^	***Mh *** **+ C** ***Mh***	**C** ***Mh *** **+ C** ***Mt***
Male cats	59	24 (40.7, 95% CI^4^ 28.1-54.3)	5 (8.5)	19 (32.2)	7 (11.9)	3 (5.1)	4 (6.8)
Female cats	87	21 (24.1, 95% CI 15.6-34.5)	10 (11.5)	13 (14.9)	1 (1.2)	3 (3.5)	0
Kitten, ≤6 months	33	4 (12.1, 95% CI 3.4-28.2)	1 (3.0)	4 (12.1)	1 (3.0)	1 (3.0)	1 (3.0)
Juvenile, >6-12 months	39	12 (30.8, 95% CI 17.0-47.6)	2 (5.1)	7 (18.0)	3 (7.7)	0	0
Young adult, >12-36 months	43	16 (37.2, 95% CI 23.0-53.3)	7 (16.3)	11 (25.6)	3 (7.0)	3 (7.0)	2 (4.7)
Adult, >36 months	31	13 (41.9, 95% CI 24.6-60.9)	5 (16.1)	10 (32.3)	1 (3.2)	2 (6.5)	1 (3.2)
Total	146	45 (30.8, 95% CI 23.5-39.0)	15 (10.3)	32 (21.9)	8 (5.5)	6 (4.1)	4 (2.7)

^1^*Mh* = *Mycoplasma haemofelis.*

^2^C*Mh* = *Candidatus* Mycoplasma haemominutum.

^3^C*Mt* = *Candidatus* Mycoplasma turicensis.

^4^95% CI = 95% confidence interval.

Microscopy of the blood smears did not reveal evidence for the presence of any pathogen, and all Knott’s tests were negative. No blood sample was PCR-positive for DNA of *A. phagocytophilum* or *L. infantum*.

The overall prevalence of haemotropic mycoplasmas was significantly higher in male compared to female cats (40.6% vs. 24.1%, p = 0.0444); however, there was no significant association of either sex to the seroprevalences of *T. gondii* (57.6% and 65.5%, respectively) and *N. caninum* (10.2% and 10.3%, respectively). Age was associated positively with the frequency of anti-*T. gondii* antibodies (p = 0.0008) and the prevalence of haemotropic mycoplasmas (p = 0.0454), but there was no association with age for the seropositivity for *N. caninum*. There was no significant difference in the prevalence of haemotropic mycoplasmas for the two seasons of collection (summer: 25/75, 33.3% vs. autumn: 20/71, 28.2%, respectively) or the status of infestation with fleas (haemotropic mycoplasma-positive/flea-positive: 17/71, 23.9% vs. haemotropic mycoplasma-positive/flea-negative: 28/75, 37.3%, respectively).

### Co-infections and exposure to multiple pathogens

A total of 45 cats (30.8%) showed evidence of exposure to and/or infection with two to four pathogens in combination (Table 5). The most common combinations were seropositivity of *T. gondii* plus mycoplasmas and seropositivity of both *T. gondii* and *N. caninum*. Overall haemotropic mycoplasma infection was not associated with the *T. gondii* status of the cats (haemotropic mycoplasma-positive/*T. gondii*-positive: 32/91, 35.2% vs. haemotropic mycoplasma-positive/*T. gondii*-negative: 13/55, 23.6%, respectively). However, sero-assessment for *N. caninum* and *T. gondii* revealed that sera yielding anti-*N. caninum* antibodies were found significantly (p = 0.0493) more frequently among the *T. gondii*-positive cats compared to the cats tested *T. gondii*-negative (*N. caninum*-positive/*T. gondii*-positive: 13/91, 14.3% vs. *N. caninum*-positive/*T. gondii*-negative: 2/55, 3.6%, respectively).

**Table 5:** Single and combined seropositivity and/or infection in 146 cats from Tirana, Albania

	**Prevalence, total (%)**
Single seropositivity or infection in cats	61 (41.8)
*Toxoplasma gondii*^*1*^	50 (34.2)
*Neospora caninum*^*1*^	1 (0.7)
*Bartonella henselae*	1 (0.7)
*Mycoplasma haemofelis*	3 (2.1)
*Candidatus* Mycoplasma haemominutum	5 (3.4)
*Candidatus* Mycoplasma turicensis	1 (0.7)
Combined seropositivity and/or infection in cats	45 (30.8)
*T. gondii* + *N. caninum*	5 (3.4)
*T. gondii* + *Leishmania infantum*^2^	1 (0.7)
*T. gondii* + *Anaplasma phagocytophilum*^1^	3 (2.1)
*T. gondii* + *M. haemofelis*	5 (3.4)
*T. gondii* + *Candidatus* M. haemominutum	12 (8.2)
*T. gondii* + *Candidatus* M. turicensis	2 (1.4)
*M. haemofelis* + *Candidatus* M. haemominutum	2 (1.4)
*Candidatus* M. haemominutum *+ Candidatus* M. turicensis	1 (0.7)
*T. gondii* + *N. caninum* + *M. haemofelis*	1 (0.7)
*T. gondii* + *N. caninum* + *Candidatus* M. haemominutum	5 (3.4)
*T. gondii* + *N. caninum* + *Candidatus* M. turicensis	1 (0.7)
*T. gondii* + *M. haemofelis* + *Candidatus* M. haemominutum	3 (2.1)
*T. gondii* + *Candidatus* M. haemominutum + *Candidatus* M. turicensis	2 (1.4)
*N. caninum* + *M. haemofelis* + *Candidatus* M. haemominutum	1 (0.7)
*T. gondii* + *N. caninum* + *Candidatus* M. haemominutum + *Candidatus* M. turicensis	1 (0.7)

^1^Seropositivity, IFAT titre ≥1:100.

^2^Seropositivity, IFAT titre ≥1:64.

## Discussion

This is the first study documenting seroprevalence of *T. gondii*, *N. caninum*, *L. infantum* and *Anaplasma* spp*.*, and the molecular detection and prevalence of *B. henselae* and three haemotropic mycoplasmas in cats in Tirana, Albania.

### Toxoplasma gondii

*Toxoplasma gondii* is one of the most prevalent cosmopolitan parasites, which can infect a wide spectrum of warm-blooded vertebrates. Based on its zoonotic nature, toxoplasmosis is one major public health issue worldwide and thus monitored closely in human medicine, but it is also considered as an important cause of reproductive disease in small ruminants. Felids, including domestic cats, are the only definitive hosts of this parasite. As cats are shedding *Toxoplasma* oocysts only for a short period, sero-surveys are suitable measures to study the epidemiology of the pathogen, and they can be used as an indicator of the environmental contamination [17-19]. Toxoplasmosis is widespread in south-east Europe [20]. The average prevalence of IgG antibodies was reported to be approximately 50% in pregnant woman in Albania [21] and among Albanians who migrated to Italy [22] and this percentage of exposure represents the upper limit of the range of currently reported data from the Balkans [23-25]. The overall seroprevalence of 62.3% in the cats from Tirana is among the highest infection percentage observed in Europe where *T. gondii* antibodies were detected in up to 70% of the cats [26]. Recently seroprevalences of 47% to 81% were reported in cats in Romania [27,28] or of almost 48% in Hungary [29]. Consistent with several other studies, the *T. gondii* seroprevalence in the cats from Tirana was positively associated with the age of the cats [28,29]. The association between seropositivity and gender, however, is unclear: in most surveys, including this study, the overall prevalence of infection in male and female cats did not differ significantly [27,28]; but there are some studies that found *T. gondii* exposure significantly more often among female cats [29,30]. The high prevalence of *T. gondii* antibodies in the cats from Tirana, Albania, is certainly related to their origin from suburban habitats with constant access to the outdoors, which has been identified as a risk factor of the infection [28]. The prevalence of antibodies to *T. gondii* in pregnant women in Albania was similar to the proportion observed in other parts of Europe. Beside consumption of undercooked meat, ‘direct soil contact’ has been thought to be associated with the seropositivity [21]. Therefore, given the high percentage of exposure to *T. gondii* in cats from Tirana, the seroprevalence of *T. gondii* in cats should be monitored further as it gives an indication of the contamination of the environment with oocysts and thus the potential implication for public health. Furthermore, the public should be informed about the risks of undercooked meat consumption.

### Neospora caninum

*Neospora caninum* is primarily associated with dogs and cattle, and neosporosis continues to be an important cause of abortion in cattle. Felines belong to the wide spectrum of domestic and wild animals that are exposed to *N. caninum*, but neither *Neospora* have been isolated from them nor were clinical signs of disease reported [31]. For Albania, there is no information on the prevalence of *N. caninum* in cattle, but a recently conducted study estimated a seroprevalence of 18.3% in dogs (Hamel et al., unpublished). Sero-surveys in cats indicated 0 to almost 25% prevalence in domestic cats [29,32-36]. The seroprevalence observed in the Tirana cats was thus in the range of results of other surveys, and consistent with a study from Italy [32].

Seropositivity was not associated to the age or sex of the cats. However, other studies found a positive correlation of the anti-*N. caninum* antibody prevalence and the cats’ age [33,35,36]. As indicated through the significant association of positive samples for *N. caninum* and *T. gondii* antibodies in this study (including the considerable number of cats with borderline anti-*N. caninum* titres) and similar observations in other studies [34,35], serological cross-reactions between these two closely related apicomplexan protozoa cannot be ruled-out because *N. caninum* and *T. gondii* share common antigens [37].

### *Anaplasma* spp.

None of the blood smears were positive for morulae, and no *A. phagocytophilum-*DNA was detected in any blood sample, but exposure to *Anaplasma* infection has been demonstrated through low titres in the serum of three cats (2.1%) using the commercial *A. phagocytophilum-*IFAT. Similar results were obtained previously when cat sera from Spain were tested [38,39]. In contrast, 40% of 30 dogs from Tirana tested positive with an *A. phagocytophilum*-IFAT [4], and in a recently completed study with 602 dogs under veterinary care from Albania, a seropositivity of 24% was established (Hamel et al.*,* unpublished). The main vector of *A. phagocytophilum* in Europe, the tick *Ixodes ricinus*, is abundant in Albania and has been recorded on dogs in the city and district of Tirana in the past [40]. However, recently conducted surveys revealed *R. sanguineus* as the predominating species of ticks parasitizing dogs from Tirana while *I. ricinus* was recovered from single animals in low numbers only [6]. On cats from Tirana, however, no *I. ricinus* at all, but few *R. sanguineus* ticks were found previously [9] as well as in the present study. The occurrence of two anaplasmas, *A. phagocytophilum* and *A. platys*, has been confirmed in blood samples of dogs from Albania with the latter species dominating (Hamel et al.*,* unpublished). *A. platys* is thought to be transmitted by *R. sanguineus*[41] and feline *A. platys* infections have been reported previously [42]. Cross-reactivity of serum samples between *A. phagocytophilum* and *A. platys* antigens have been described in dogs [43-45]. Thus, seropositivity detected with the commercial *A. phagocytophilum*-IFAT in the cats in this study may be attributed to infection with either of the two or both anaplasmas.

### Leishmania infantum

Albania belongs to the countries where zoonotic visceral leishmaniosis is endemic, and the disease is of great relevance in humans [46-48]. Competent vectors of *L. infantum*, the causative agent of human and canine leishmaniosis in Albania [49], are abundant in the country [50], and their occurrence was also documented for the district and city of Tirana [51]. Sero-surveys conducted in Albania indicated average anti-*Leishmania* antibody prevalences of 4–5.1% in dogs of different categories and geographic origin [3] (Hamel et al.*,* unpublished). In contrast to the situation in dogs, feline leishmaniosis occurs sporadically and usually only in regions where canine leishmaniosis is endemic. The recently proven infectiousness of cats to sand-flies re-emphasized the question of the role of felines in the epidemiology of leishmaniosis which is still not clear [52,53]. With only 1 out of 146 feline sera from Tirana testing as *Leishmania*-positive, the percentage of exposure of cats to the parasite in the present study was at the lower end of the wide range of findings reported from other countries in the south of mainland Europe [54-58] and is much lower than the 5.1% seroprevalence recorded in dogs from Tirana (Hamel et al., unpublished). For the Balkans, information on feline leishmaniosis is available apparently through two recently conducted sero-surveys in Greece only. For both studies, serum samples were collected from stray cats in the Thessaloniki area, but the seroprevalences determined differed substantially with 21.6% [59] or 3.9% [60], respectively.

### Dirofilaria immitis

Canine *D. immitis* is endemic in Albania [3-5,7], and several of its incriminated vectors are common representatives of the culicid fauna in the country [61]. However, microfilariae were not detected in any blood smears or by using the Knott’s test neither was heartworm antigen detected in any serum sample of the cats from Tirana. Although canine heartworm infection was reported with increasing frequency from most of the countries of the Balkans in the recent past [7,62], the authors are not aware of any survey on feline dirofilariosis in the region. Similar to leishmaniosis, feline dirofilariosis is much less frequently diagnosed compared to canine dirofilariosis in a given region and, based on data collected in endemic areas, the prevalence of heartworm infection in cats can be expected to be 5% to 10% of that seen in dogs [63].

### Bartonella henselae

As the main reservoir for *B. henselae*, the cat is a host which is often associated with *Bartonella* infections and with their transmission to man, while fleas have been proven to serve as vectors for transmission of the agents among cats [64,65]. Human disease associated to infection with *Bartonella* species as well as seropositivity to *Bartonella* spp. has been described from some countries in the Balkans [66,67] but yet not from Albania. The prevalence of *Bartonella* infection in cats in the Balkan region is largely unknown. To the knowledge of the authors, there is only a small-scale survey from Serbia, which found that 57% of 40 cats had anti-*B. henselae* antibodies [68]. *Bartonella henselae* and *B. clarridgeiae* were demonstrated in *C. felis* fleas collected from seven cats from Tirana [10] including five cats studied in this survey. The single flea collected from the four months old, female kitten whose blood tested positive for *B. henselae* in the present study, however, did not harbour *Bartonella-*DNA [10]. In contrast, the positive fleas reported by Silaghi et al. (2012) were collected from cats negative for *Bartonella* spp. [10]. Thus, further studies are needed to understand the involvement of cats in the epidemiology of *Bartonella* infections in the Balkans.

### Haemotropic mycoplasmas

Four haemotropic mycoplasmas have been recognized in cats: *M. haemofelis*, *Candidatus* M. haemominutum, *Candidatus* M. turicenis and *Candidatus* M. haematoparvum-like. These mycoplasmas differ in their pathogenicity, with *M. haemofelis* being the most virulent one that may cause severe haemolytic anaemia. The others may cause changes in blood parameters, but rarely cause diseases without concurrent infections; for the latter one no data is available. Subclinical infections with cats acting as carriers with subclinical infection are common. Feline haemotropic mycoplasma infections are believed to have a worldwide distribution with variable prevalence in the different populations of cats [69,70]. For Albania, this is the first study to document the occurrence and prevalence of three haemotropic mycoplasmas in cats, and it is the only other study on feline haemotropic mycoplasma infection from the Balkans beside one report from Greece [71]. The overall proportion of haemotropic mycoplasma infection of almost 31% as well as the prevalence of the individual mycoplasmas, including the percentage of co-infections, were within the range of results obtained in other studies in Europe [71-75]. The highest overall prevalence of haemotropic mycoplasmas in Europe (43.4%) was found in a recently reported study from Portugal, where all four feline haemotropic mycoplasmas were identified, with all four mycoplasmas co-infecting one cat [76]. In line with some other studies, male sex [69,71,72] and older age [16,71,77,78] were found significantly associated with the detection of haemotropic mycoplasma DNA in the cats from Tirana. Contradictory to Gentilini et al. (2009), who suggested an association of summer season and higher prevalence of feline haemotropic mycoplasma infection because of a higher ectoparasite load which facilitates vectorial transmission of infections, own data and results of studies from Germany and Portugal [72,76,79] do not indicate a positive correlation of the presence of blood-feeding arthropods and feline haemoplasmosis. Furthermore, analysis for mycoplasma DNA of all fleas collected from the cats in this study revealed only DNA of *Candidatus* M. haemominutum in two single *C. felis* fleas from two cats (unpublished data). One of those cats was a kitten that tested positive for *B. henselae* but was negative for haemotropic mycoplasma infection and had one flea only; the other cat was infected with *Candidatus* M. haemominutum and had three fleas. Both, the lack of correlation of the presence of blood-feeding arthropods and detection of DNA of haemotropic mycoplasmas and the marked discrepancy between the detection percentages of haemotropic mycoplasmas in the cats and in their fleas support hypotheses of the existence of routes of transmission of these bacterial organisms other than fleas acting as vectors. Aggressive interaction, such as cat bites, has been suggested as the possible route of transmission and this is supported by the higher prevalence in older and male cats seen in the present study [70].

## Conclusion

In conclusion, with the broad screening panel including direct and indirect methods applied in the present study, a wide spectrum of parasitic or vector-borne agents and/or exposure to them could be detected in Albanian cats from Tirana.

## Competing interests

The authors declare that they have no competing interests.

## Authors’ contributions

MK, DR, IK and ES collected the samples. CS performed the laboratory examinations. CS, MK and SR analysed the data. CS and SR drafted the manuscript, which was critically revised by MK, DH and KP. All authors read and approved the final version of the manuscript.
